# Supplementary material for: A Low-Cost Modular Imaging System for Rapid, Multiplexed Immunofluorescence Detection in Clinical Tissues
Source: Int J Mol Sci. 2023 Apr 10;24(8):7008. doi: 10.3390/ijms24087008 (PMC10138925; doi:10.3390/ijms24087008)
Supplement: Supplementary file 1 [file ijms-24-07008-s001.zip › ijms-2275499-supplementary.pdf]

A LOW-COST MODULAR IMAGING SYSTEM FOR RAPID, MULTIPLEXED IMMUNOFLUORESCENCE DETECTION IN CLINICAL TISSUES

Joshua Gu[1,2], Hannah Jian[3], Christine Wei[4], Jessica Shiu[5], Anand Ganesan[1,5,6], Weian Zhao[1,2,4,6,7,8,9,*], Per Niklas Hedde[10,*]

1. Department of Biological Chemistry, University of California, Irvine, California 92697, United States
2. Sue and Bill Gross Stem Cell Research Center, University of California, Irvine, California 92697, United States
3. Department of Molecular Biology and Biochemistry, University of California, Irvine, California 92697, United States
4. Department of Pharmaceutical Sciences, University of California, Irvine, California 92697, United States
5. Department of Dermatology, University of California, Irvine, CA, USA.
6. Chao Family Comprehensive Cancer Center, University of California, Irvine, California 92697, United States
7. Edwards Life Sciences Center for Advanced Cardiovascular Technology, University of California, Irvine, CA, 92697, United States
8. Department of Biomedical Engineering, University of California, Irvine, CA, 92697, United States
9. Institute for Immunology, University of California, Irvine, CA 92697, United States
10. Beckman Laser Institute and Medical Clinic, University of California, Irvine, California 92697, United States

*Corresponding authors: phedde@uci.edu, weianz@uci.edu

Supplementary Materials

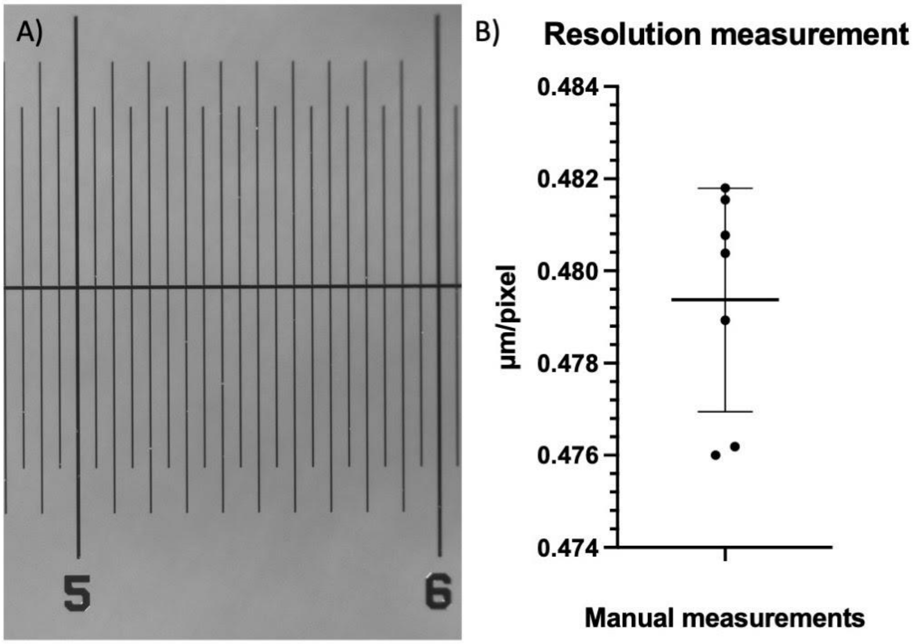

Supplementary Figure S1: Resolution calibration for Tissue Imager
A) Image of the Thorlabs 10 mm ruler with 50 µm spacing between the divisions B) Measurements plotted with an average manual measurement (n = 7) of 0.48 µm/pixel on the Thorlabs 10 mm ruler. Scatter plot show the average and standard deviation.

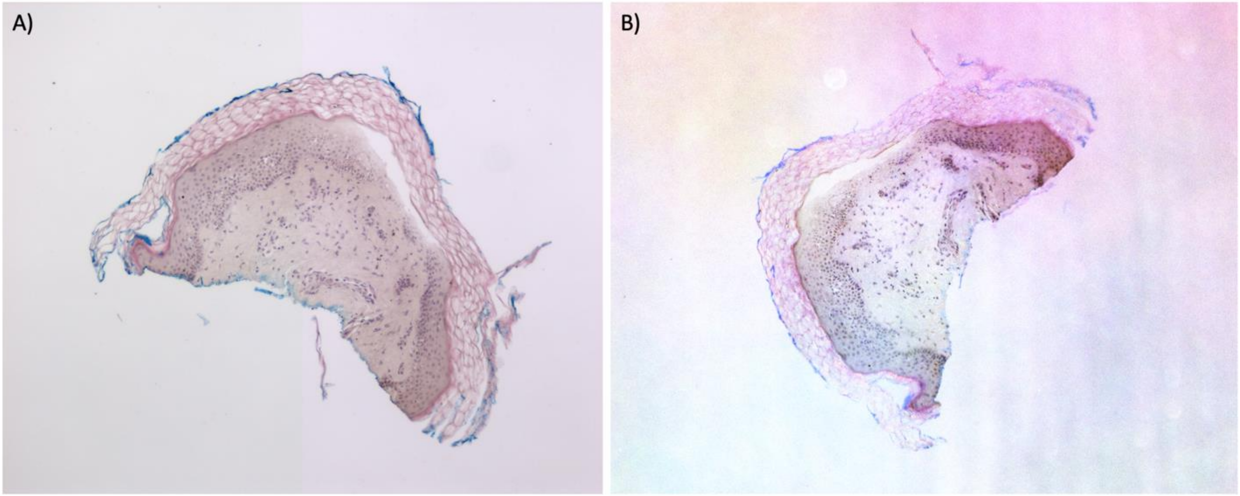

Supplementary Figure S2: Brightfield image on Tissue Imager with RGYB imaging
H&E staining on human CTCL skin FFPE tissue. A) Imaged on Nikon Eclipse E400 microscope. B) RGYB imaging on Tissue Imager.

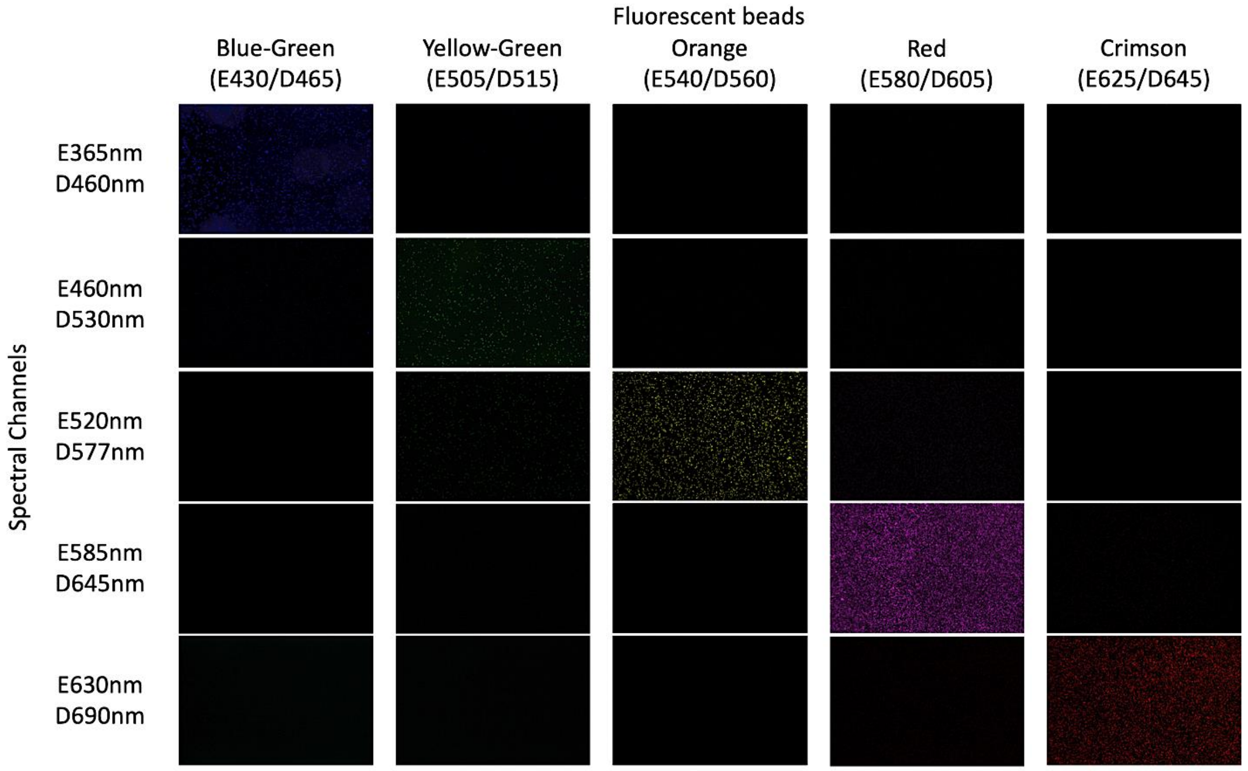

Supplementary Figure S3: Fluorescence bead and spectral channel calibration for Tissue Imager
Each 1 µm fluorescent bead population (column) was imaged in each spectral channel on the Tissue Imager (row) to validate the absence of crosstalk. The beads were Blue-Green (E430nm/D465nm), Yellow-Green (E505nm/D515nm), Orange (E540nm/D560nm), Red (E580nm/D605nm), and Crimson (E625nm/D645nm).

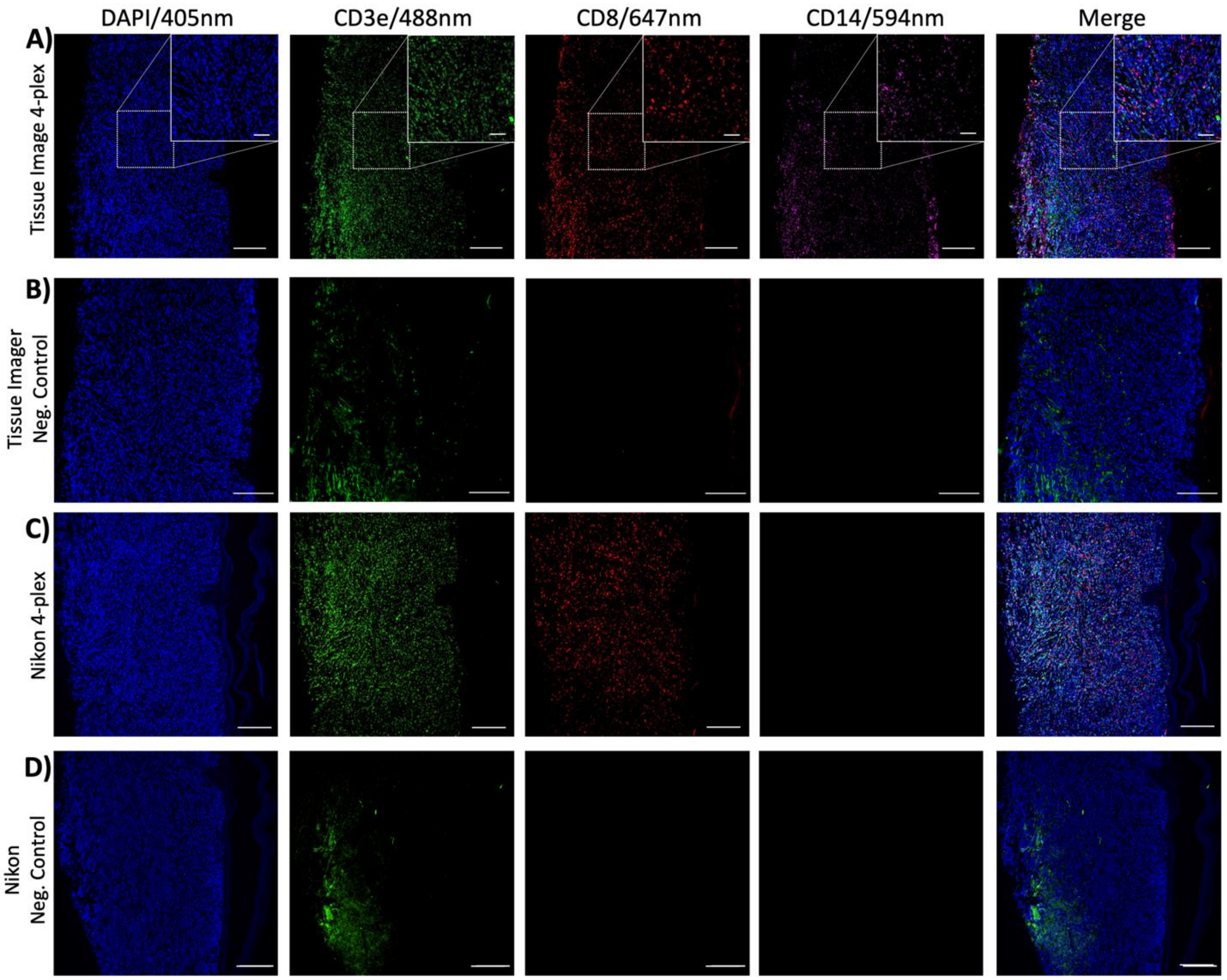

Supplementary Figure S4: 4-plex panel with corresponding controls

A) The 4-plex detection of DAPI, CD3e, CD8, CD14 in human CTCL skin FFPE tissue imaged on the Tissue Imager. B) The negative control of secondary fluorophore antibody staining only on imaged on the Tissue Imager. C) The 4-plex detection of DAPI, CD3e, CD8, CD14 in human CTCL skin FFPE tissue imaged on the Nikon microscope. B) The negative control of secondary fluorophore antibody staining only on imaged on the Nikon microscope. Scale bar, 200 µm.

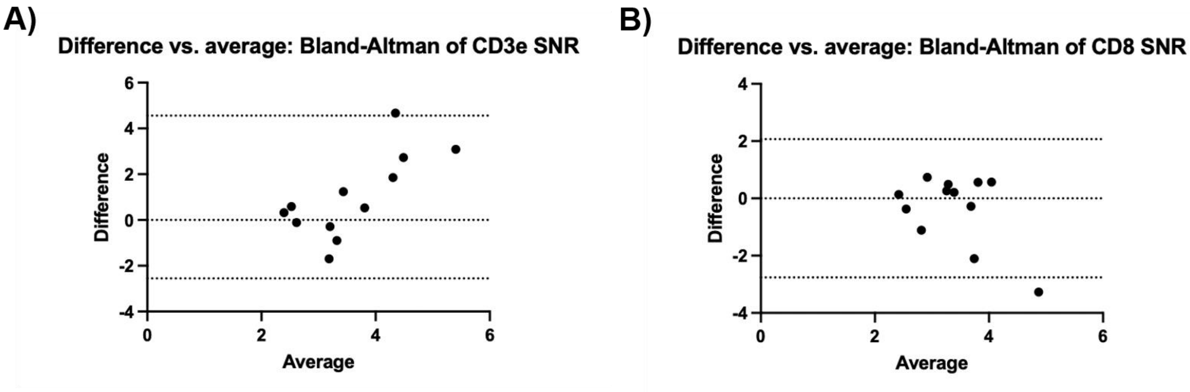

Supplementary Figure S5: Bland-Altman plots of the SNR differences between the Tissue Imager and the Nikon microscope
A) SNR difference of CD3e and B) SNR difference of CD8.

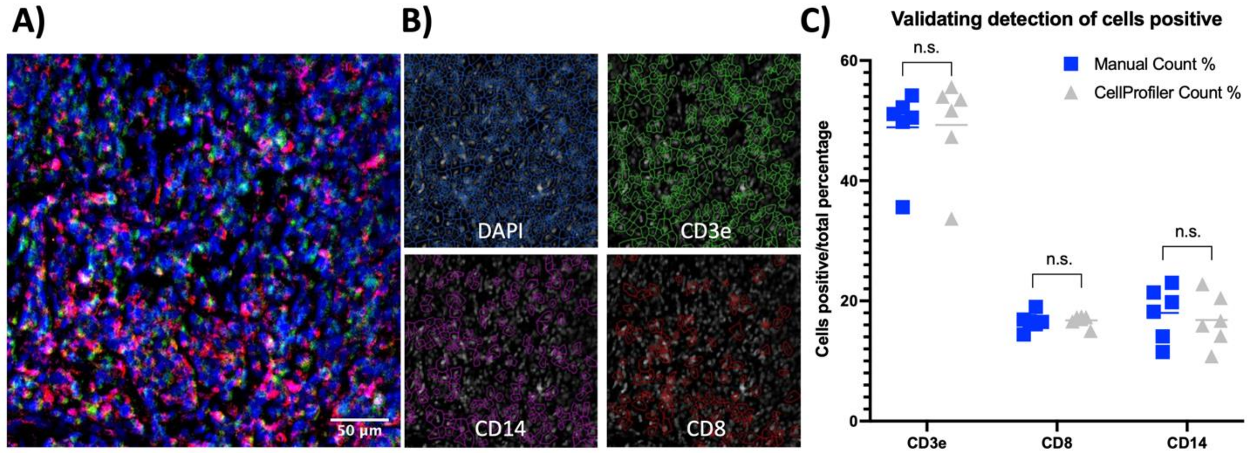

Supplementary Figure S6: Manual counting to validate CellProfiler detection
A) Cropped 700 x 700 pixel merged image of 4-plex (DAPI, CD3e, CD8, CD14) on human skin cutaneous T-cell lymphoma FFPE tissue. B) CellProfiler outline of detected cells with DAPI, CD3e, CD8, and CD14. C) Plot comparing manual counts to CellProfiler counts of CD3e, CD8, and CD14 in cropped images (n = 6). The mean was plotted as a line. Pairwise T-test between manual counting and CellProfiler counting for each marker was not significant (P = 0.7549, 0.8246, 0.2237 for CD3e, CD8, CD14 respectively).

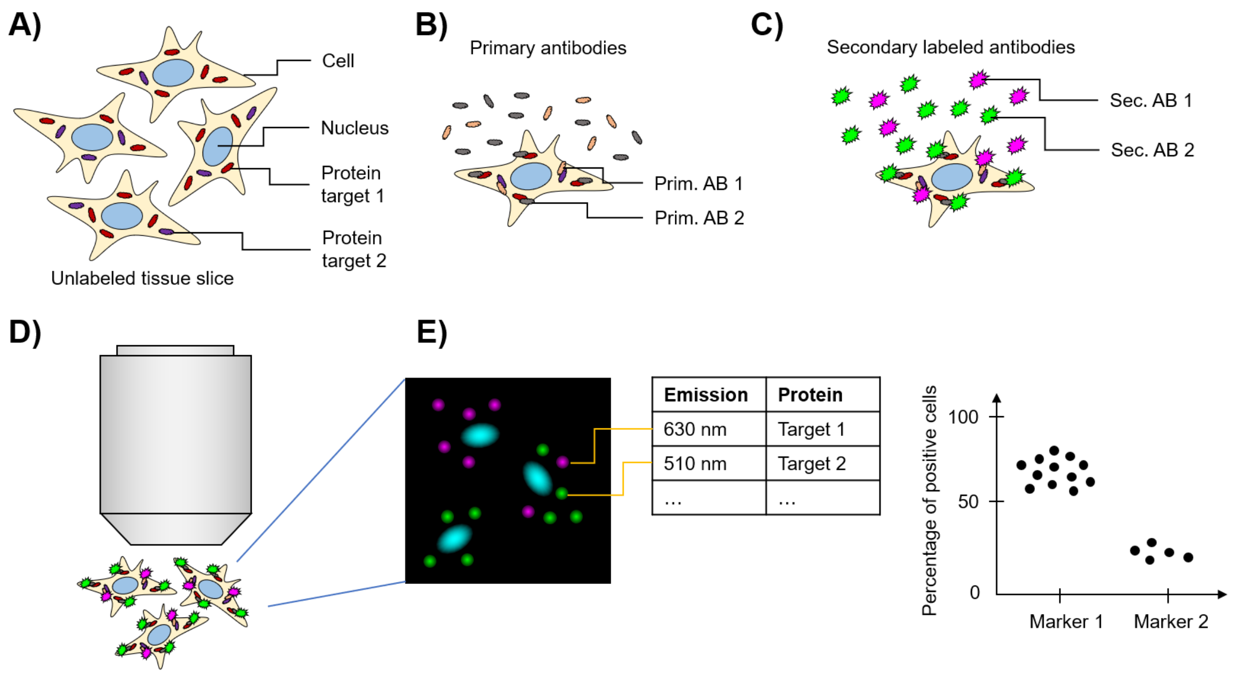

Supplementary Figure S7: Workflow schematic overview

A) Sample(s) can be tissue slices or fixed cells, proteins of interest are targeted for detection. B) Primary antibodies are used to target the proteins of interest. C) Secondary fluorescently labeled antibodies are added to bind to the respective primary probes. D) Labeled targets are imaged with the Tissue Imager to interrogate the spectral characteristics of the labeled moieties that are used to E) identify which fluorophore labels are present in each puncta thus cellular distributions of the present protein targets can be analyzed in a multiplexed fashion.
